# Identification and Characterization of Key Genes for Nitrogen Utilization from *Saccharum spontaneum* Sub-Genome in Modern Sugarcane Cultivar

**DOI:** 10.3390/ijms26010226

**Published:** 2024-12-30

**Authors:** Qianlong Hui, Ting Song, Dantong Yang, Qibin Wu, Jinlong Guo, Youxiong Que, Liping Xu

**Affiliations:** 1 National Engineering Research Center for Sugarcane, College of Agriculture, Fujian Agriculture and Forestry University, Fuzhou 350002, China; huiqianlong@163.com (Q.H.); songting2024@163.com (T.S.); dantong0903@163.com (D.Y.); wqbaidqq@163.com (Q.W.); jlguo@fafu.edu.cn (J.G.); 2 National Key Laboratory for Tropical Crop Breeding, Institute of Tropical Bioscience and Biotechnology/Sanya Research Institute, Chinese Academy of Tropical Agricultural Sciences, Sanya 572024, China

**Keywords:** sugarcane, nitrogen use efficiency, genetic mechanisms, sub-genome, *ScNRT2.3*

## Abstract

Sugarcane (*Saccharum* spp.) is globally considered an important crop for sugar and biofuel production. During sugarcane production, the heavy reliance on chemical nitrogen fertilizer has resulted in low nitrogen use efficiency (NUE) and high loss. Up until now, there has been extensive research on the transcriptomic dynamics during sugarcane response to low nitrogen (LN) stress. However, the specific contribution of *S. spontaneum* to the NUE of modern sugarcane remains unclear. In the present study, the comparative transcriptome analysis of two contrasting sugarcane cultivars in response to nitrogen deficiency was performed via the combination of genomes of *S. spontaneum* and *S. officinarum*. Sub-genome analysis indicated that *S. spontaneum* supports the high NUE of modern sugarcane by providing genes related to nitrogen and carbohydrate metabolism, photosynthesis, and amino acid metabolism. Additionally, the key genes involved in nitrogen metabolism from the *S. spontaneum* were successfully identified through weighted gene co-expression network analyses (WGCNA), and a high-affinity nitrate transporter named *ScNRT2.3* was subsequently cloned. Heterogeneous expression of the *ScNRT2.3*, a cell membrane-localized protein, could enhance the growth of *Arabidopsis* under low nitrate conditions. Furthermore, a conserved protein module known as NAR2.1/NRT2.3 was shown to regulate the response to LN stress in sugarcane roots through molecular interaction. This work helps to clarify the contribution of *S. spontaneum* to the NUE in modern sugarcane, and the function of the *ScNRT2.3* in sugarcane.

## 1. Introduction

In plants, nitrogen (N) acts as a macro- and essential mineral nutrient, playing a vital role in growth and development [1,2]. Furthermore, N functions as a signaling molecule that influences plant morphology, various biological processes, and gene expression [3]. Sugarcane (*Saccharum* spp.) is a significant crop for sugar, biofuel, and fodder production, with high nutrient requirements [4,5]. Due to its long growth cycle and high biomass production, N is a critical nutrient for sugarcane fertilization. China, as the world’s third-largest sugarcane and sugar producer, faces challenges from the rapid depletion of soil nutrients due to continuous sugarcane cultivation [6]. To achieve high yields, farmers in southern China often resort to excessive chemical N fertilizer applications, about 400–800 kg N per hectare, which is three times the global average [7,8]. However, only a fraction of the applied N (20–40%) is absorbed and utilized by sugarcane [9]. The excess N application and low use efficiency raise significant environmental concerns, such as those related to water pollution, soil acidification, and ecosystem degradation [10]. The development and adoption of new cultivars with high nitrogen use efficiency (NUE) represents an effective strategy to enhance fertilizer use efficiency, increase yields, and mitigate environmental degradation [11]. Nonetheless, achieving this objective requires a thorough understanding of the processes involved in plant N uptake and assimilation, particularly under low nitrogen (LN) stress conditions.

NUE is a complex trait influenced by various biological processes including nitrogen uptake, assimilation, translocation, and re-mobilization in plants [12,13]. These responses are contingent upon factors such as genotype, environmental conditions, nitrogen availability, and plant age. Numerous genes contribute to NUE either independently or through interactions with other genes or the environment [13]. The identification of key regulatory genes and the comprehension of their mechanisms present significant challenge. Plants can absorb and utilize two forms of inorganic nitrogen (NH_4_^+^ and NO_3_^−^) from the soil directly, facilitated by various transporters and assimilation enzymes such as nitrate transporter 1/peptide transporter family (NPF), nitrate transporter 2 family (NRT2), nitrate reductase (NIA), nitrite reductase (NIR), ammonium transporters (AMTs), glutamine synthetase (GS), glutamate synthase (GOGAT), and glutamate dehydrogenase (GDH) [12,13]. Recent advancements in comparative transcriptome, comparative genome, and the integration of comparative transcriptome and genome analyses, have shed light on the molecular mechanisms regulating plant NUE. Zhang et al. [14] identified a specific regulatory network and a hub gene (*CsAMT1.2*) responsive to LN treatment in tea plants through comparative transcriptome analysis. Neeraja et al. [15] discovered that the downregulation of certain less critical metabolic pathway genes in N-efficient rice genotypes serves as a strategy of acclimation to N deficiency by comparative transcriptome analysis. Wang et al. [16] demonstrated that the nodule-inception-like protein 6 (*ZmNLP6*) gene associated with alternative splicing events in response to LN stress plays a crucial role in N starvation by the combination of transcriptome and DNA affinity purification sequencing (DAP-seq) analyses.

Modern sugarcane cultivars (*Saccharum* spp. hybrids) are predominantly bred by crossing *S. officinarum* and *S. spontaneum*, inheriting high sugar content from *S. officinarum* and excellent stress tolerance from *S. spontaneum* [17]. N deficiency is one type of stress, but whether the tolerance of sugarcane to this stress comes from the contribution of *S. spontaneum* remains unclear. The complexity and highly polyploid nature of sugarcane present a significant challenge in genome assembly compared to other crops [18,19], and hinder advancements in understanding molecular mechanisms related to N utilization and enhancing NUE through genetic modifications in sugarcane. In recent years, several reference genomes of sugarcane have been published, including three from *S. spontaneum* [18,20,21], one from *S. officinarum* [22], and one from modern sugarcane cultivars assembled at the scaffold level [23], and three from modern sugarcane cultivars assembled at the chromosome level [19,24,25]. However, the genome of the elite sugarcane parent ROC22 with high NUE is still un-assembled, and it has a high proportion of chromosomes derived from interspecific recombination between *S. officinarum* and *S. spontaneum* [26]. As a result, the de novo assembly was selected as a commonly used approach for the transcriptomic analysis of sugarcane hybrids. In our previous study, a comparative transcriptome analysis was conducted between two NUE-contrasting cultivars with different genetic backgrounds under LN stress conditions based on de novo assembly [27], while the absence of data on allelic genes, alternative splicing events, and sub-genomes significantly restricted the depth of insights that could be obtained from these analyses. A recent study examined the contribution of the *S. spontaneum* sub-genome to sugar accumulation in varieties with different sugar contents by integrating the genomes of *S. spontaneum* AP85-441 and *S. officinarum* LA-purple, and shed light on the mechanisms behind high-sugar cultivars and the role of the *S. spontaneum* sub-genome in enhancing sugar accumulation in modern sugarcane hybrids [28]. In this study, we re-analyzed the RNA-seq raw data from our previous work by utilizing the reference genomes of *S. spontaneum* NP-X and *S. officinarum* LA-purple. Our goal was to investigate how genes from the sub-genome of *S. spontaneum* in modern sugarcane cultivar ROC22 contribute to its high NUE via comparative transcriptome analysis and weighted gene co-expression network analysis (WGCNA). Additionally, we identified and characterized a hub gene *ScNRT2.3* derived from the sub-genome of *S. spontaneum* in ROC22. Furthermore, a conserved functional module between ScNRT2.3 and ScNAR2.1 was confirmed by yeast two-hybrid (Y2H), bimolecular fluorescence complementation (BiFC) and Co-Immunoprecipitation (CoIP) assays. These findings provide valuable insights into the biological function of *ScNRT2.3* and lay a solid foundation for understanding the molecular mechanisms underlying NUE in sugarcane.

## 2. Results

### 2.1. High-Quality Transcriptome Assembly of ROC22 Was Obtained Based on the Combined Genome

**Figure 1:**
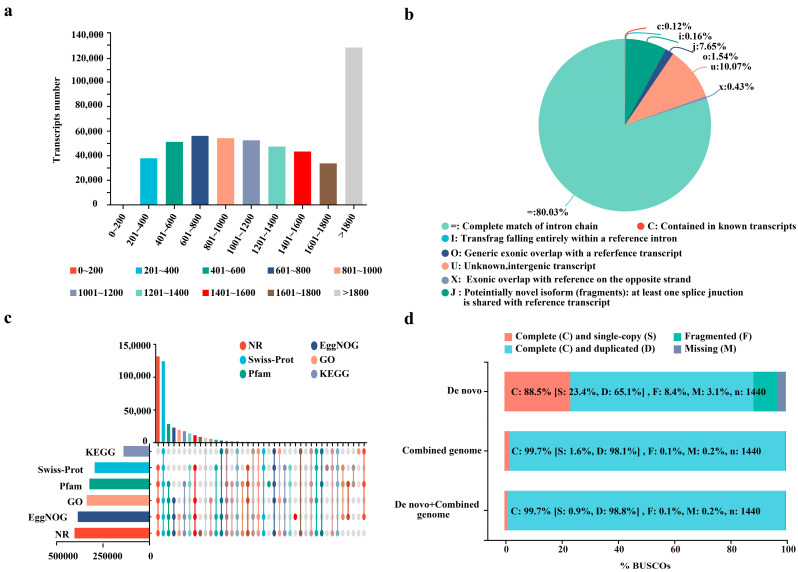
The annotation and evaluation of the assembled transcripts and genes. (**a**) The length distribution of the assembled transcripts. (**b**) The classification of the assembled transcripts. (**c**) The annotation of the genes using KEGG, Swiss-Prot, Pfam, GO, EggNOG, and NR databases. (**d**) The assessment of the assembly integrity among various assembly strategies by using homologous single-copy conserved genes.

As Table S1 shows, the alignment efficiency of the total alignment ranged from 71.01% to 83.85%, with multiple mapped ratios ranging from 41.66% to 49.97% and uniquely mapped ratios from 29.35% to 37.00% for each sample using the combined genome sequences. After reference-based assembly, a total of 501,969 transcripts (402,326 from the combined genome and 99,643 from the assembly) were successfully obtained, coding 421,860 genes (376,376 from the combined genome and 45,484 from the assembly). The length distribution of assembled transcripts was illustrated in Figure 1a. Among them, transcripts with a length of more than 1800 bp account for the most (25.44%), while those with a length of 0-200 bp account for the least (0.02%). The assembled transcripts were categorized into six types based on their sequence characteristics, as follows: including known transcripts (603, 0.12%), transfrag falling entirely within a reference intron (825, 0.16%), potential novel isoform (fragment), at least one splice junction is consistent with reference transcripts (38,409, 7.65%), generic exonic overlap with a reference transcripts (7718, 1.54%), unknown or intergenic transcript (50,538, 10.07%), and exonic overlap with reference on the opposite strand (2153, 0.43%) (Figure 1b). The function annotation results indicate that 95.54% (403,060 out of 421,860) of the genes were properly annotated across various databases, such as GO (79.95%, 337,291 genes), KEGG (32.94%, 138,977 genes), COG (91.29%, 385,132 genes), NR (95.48%, 402,788 genes), Swiss-Prot (69.72%, 294,122 genes), and Pfam (76.46%, 322,554 genes) (Figure 1c). Benchmarking Universal Single-Copy Orthologs (BUSCOs) analysis revealed a relatively complete assembly, with 99.7% (n = 1436, consisting of 1423 complete and duplicated BUSCOs and 13 complete and single-copy BUSCOs) of BUSCOs being full sequences, only 0.1% (*n* = 1) being fragmented sequences, and 0.2% (*n* = 3) being absent in the assembly within the embryophyta lineage (Figure 1d). The high function and BUSCOs annotation ratios indicate that the combined genome and reference-based assembly strategies were appropriate for our transcriptome analysis.

### 2.2. The Sub-Genome of S. spontaneum Is Responsible for the High NUE of ROC22

**Figure 2:**
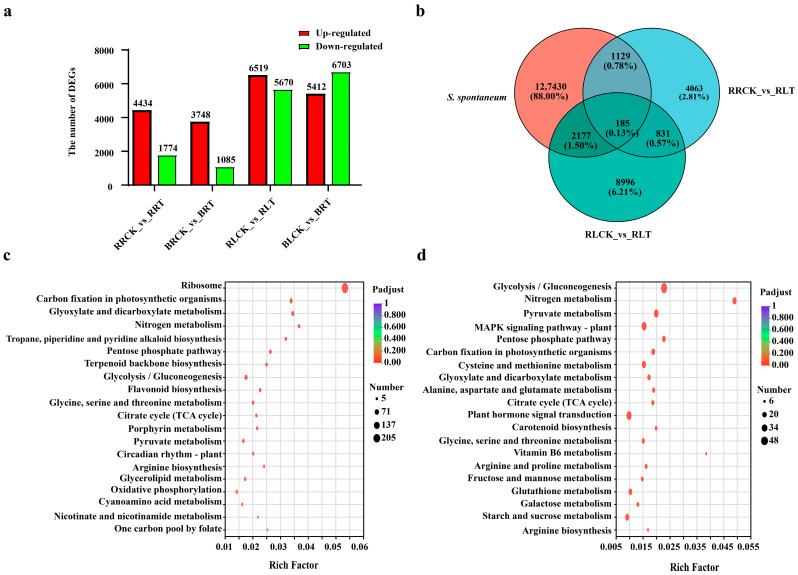
Identification and function annotation of *S. spontaneum* genes associated with LN stress response in ROC22. (**a**) The DEGs in ROC22 and Badila. The first letters R and B represent the varieties ROC22 and Badila, respectively; the second letters L and R represent leaf and root, respectively. The letters CK and T represents normal- and low-nitrogen treatment, respectively. (**b**) The *S. spontaneum* genes involved in LN stress response in ROC22. (**c**,**d**) represents the top 20 KEGG pathways in ROC22 leaves and roots, according to the *S. spontaneum* genes, respectively.

To evaluate the contribution of *S. spontaneum* to the modern sugarcane cultivar, the differential expression analysis was used to identify the DEGs from *S. spontaneum* in ROC22. As Figure 2a,b shows, there were 6519 (5359 from *S. officinarum* and 1160 from *S. spontaneum*) up-regulated and 5670 (4468 from *S. officinarum* and 1202 from *S. spontaneum*) down-regulated DEGs in the leaf tissues of ROC22, while there were 5412 (from *S. officinarum*) up-regulated and 6703 (from *S. officinarum*) down-regulated DEGs in Badila leaves (Table S2). The situation in the roots, however, was different, with leaves between ROC22 and Badila. In detail, a total of 6208 DEGs, including 4434 (3508 from *S. officinarum* and 926 from *S. spontaneum*) up-regulated and 1774 (1386 from *S. officinarum* and 388 from *S. spontaneum*) down-regulated DEGs, were identified from the root tissues of ROC22 (Figure 2a,b, Table S2). The DEGs identified from the root tissues of Badila numbered 3748 (from *S. officinarum*) up-regulated and 1085 (from *S. officinarum*) down-regulated, which numbers are significantly less than that in the roots of ROC22 (Figure 2a,b). These findings reveal that the sub-genome of *S. spontaneum* plays a vital role in the divergence of NUE between ROC22 and Badila.

In addition, a KEGG enrichment analysis was performed to investigate the contribution of *S. spontaneum* sub-genome to the high NUE of ROC22. As Figure 2c shows, the strongest enrichment pathway in leaves is ribosome, followed by N metabolism, glyoxylate and dicarboxylate metabolism, carbon fixation in photosynthetic organisms, and tropane, piperidine and pyridine alkaloid biosynthesis. Interestingly, the most abundant pathway in the leaves was also the ribosome (Table S3). Different from the leaf, however, the most significant enrichment pathway in the roots was N metabolism, followed by vitamin B6 metabolism, glycolysis/gluconeogenesis, the pentose phosphate pathway, and pyruvate metabolism (Figure 2d, Table S3). In addition, the pathway of glycolysis/gluconeogenesis was considered as the most abundant pathway in roots (Figure 2d, Table S3). The findings indicate that the *S. spontaneum* sub-genome supports the NUE of modern sugarcane cultivar by providing genes involved in nitrogen metabolism, photosynthetic carbon assimilation, carbohydrate metabolism, and amino acid metabolism pathways.

### 2.3. The Hub Modules and Genes Involved in High NUE of Cultivar ROC22

**Figure 3:**
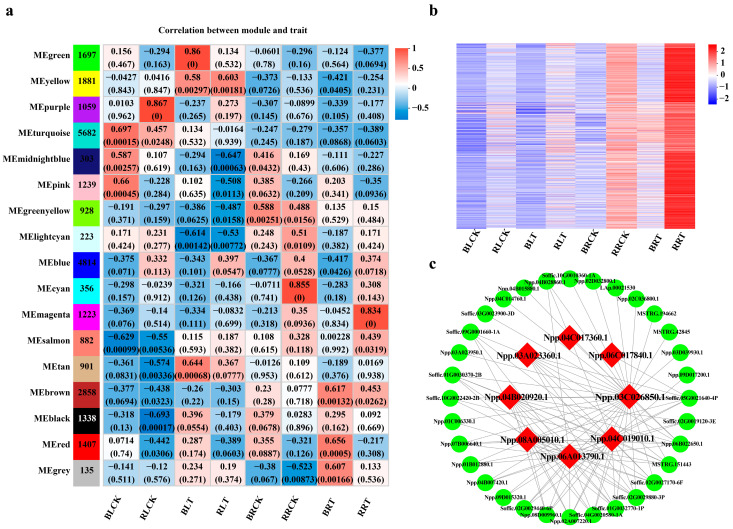
The co-expression network of the high-NUE cultivar ROC22’s specifically associated modules. (**a**) The module–trait associations revealed by Pearson correlation coefficient. The leftmost column indicates different co-expression modules. The numbers in the figure indicate the correlation between the modules and traits, and the numbers in the parentheses are the correlation *p* values. (**b**) A heatmap showing genes in the magenta module that were strongly expressed in ROC22 roots. (**c**) The identification of the gene co-expression network and hub genes. The candidate hub genes are shown in a red box.

After filtering out genes with a coefficient of variation below 0.2, the clustering tree was constructed for all eight groups with 24 samples (Figure S1). Subsequently, modules with similar expression patterns were merged using the dynamic tree-cutting method, resulting in a total of 25,401 DEGs grouped into 17 co-expressed gene modules (Figure 3a). The turquoise module contained the highest number of DEGs (5682 DEGs), followed by the blue module (4814 DEGs), brown module (2858 DEGs), yellow module (1881 DEGs), and green module. In contrast, the grey module had the fewest DEGs, totaling 135, and represented genes that did not fit into any other modules (Figure 3a). The correlation coefficients between module eigengenes and traits were examined to investigate the relationship between identified modules and the genotypes. Among the 17 modules, only 3 modules exhibited a significant and positive correlation with the high-NUE cultivar ROC22 (Figure 3a). The purple module had a uniquely significant and positive correlation with ROC22 leaves under normal nitrogen conditions (Figure 3a). The yellow module displayed a significant positive correlation with both leaf samples of ROC22 and Badila after 6 h of LN stress (Figure 3a). The cyan module demonstrated uniquely significant correlations with ROC22 roots under normal nitrogen conditions. However, the magenta module was suggested to have uniquely significant correlations with ROC22 roots after 3 h of LN treatment (Figure 3a). Therefore, the magenta module was selected as the target gene module for further analysis, based on the tissue relationship, treatment, and significance.

In the magenta module, a total of 1223 DEGs were found to show up-regulation in ROC22 roots at 3 h after LN stress, including 832 *S. officinarum* genes and 391 *S. spontaneum* genes (Figure 3a,b, Table S4). Among these, 41 (20 from *S. officinarum* and 21 from *S. spontaneum*) N uptake and assimilation genes (NAGs) were highlighted as particularly responsive to LN stress (Table S4). By analyzing the top 100 coefficient network relationships of NAGs from *S. spontaneum*, 2 *NRT2*, 1 *NAR2.1*, 3 *AMT1*, and 2 *GDH2* genes were identified as the hub genes (Figure 3c, Table 1 and Table S5).

### 2.4. Identification of ScNRT2.3 from the High NUE Sugarcane Cultivar ROC22

**Figure 4:**
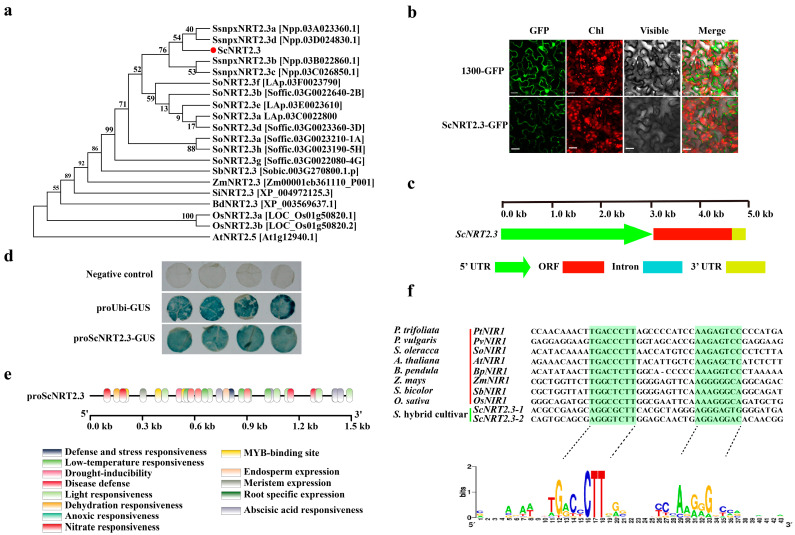
The subcellular localization of and promoter activity of *ScNRT2.3*. (**a**) The phylogenetic tree of the NRT2.3 protein between sugarcane and other plant species. The red circle indicated the NRT2.3 protein from sugarcane. (**b**) The subcellular location of the protein ScNRT2.3. Scale bars = 20 μm. (**c**) The structure schematic diagrams of *ScNRT2.3*. (**d**) The GUS activity of promoter of *ScNRT2.3*. (**e**) The distribution and functional prediction of *cis*-acting elements in the promoter of *ScNRT2.3*. (**f**) The sequence characteristics of the nitrate response element in the promoter of *ScNRT2.3*. The green markers represented potential nitrate-responsive elements.

WGCNA revealed that two genes (*Npp.03A023360.1* and *Npp.03C026850.1*) encoding high-affinity nitrate transporter 2.3 from the sub-genome of *S. spontaneum* act as the potential hub genes contributing to the difference between NUE in roots between ROC22 and Badila (Figure 3c, Table 1). To functionally characterize the gene *NRT2.3* in sugarcane, a full-length cDNA sequence of the *ScNRT2.3* was successfully cloned from the high-NUE cultivar ROC22. The full-length cDNA of *ScNRT2.3* was 1646 bp long, containing an open reading frame (ORF) of 1548 bp, a 5′-untranslated region (UTR) of 43 bp, and a 3′-UTR of 55 bp (Figure S2). The primary structure prediction results of ScNRT2.3 suggest that the number of amino acids is 515, the theoretical isoelectric point (*p*I) is 9.14, the molecular weight is 55.29 kDa, the instability index is 38.36, and the Grand average of hydropathicity (GRAVY) is 0.424. This information indicates that ScNRT2.3 is an alkaline and stable hydrophobic protein. The multiple alignment and phylogenetic analysis of NRT2.3 between sugarcane and other plant species suggests that the ScNRT2.3 is conserved and closest related with NRT2.3 proteins from *S. spontaneum*, *S. officinarum*, *Sorghum*, *Zea mays*, and *Oryza sativa* (Figure 4a and Figure S3, Table S6). The subcellular localization result shows that the GFP signal is found in the plasma membrane, indicating that ScNRT2.3 is a membrane protein (Figure 4b).

In addition, the promoter sequence was also cloned to further evaluate the potential function of *ScNRT2.3* in sugarcane. The sequencing results show that the fragment contains approximately 3022 bp of the upstream of translation initiation site (ATG) in the *ScNRT2.3* in sugarcane (Figure 4c). The results of promoter activity analysis suggest that GUS driven by the promoter of *ScNRT2.3* can be clearly observed in the leaves of *Nicotiana benthamiana*, indicating that the promoter of *ScNRT2.3* has been successfully isolated (Figure 4d). The sequence analysis shows that the *ScNRT2.3* promoter contains a diverse array of *cis*-acting elements, including plant growth and development (CCGTCC-motif, CCGTCC-box, GCN4_motif, and motif I), hormone-responsive elements (ABRE), light-responsive elements (ACE, G-Box, G-box, GATA-motif, MRE, Sp1TCT-motif), stress-responsive elements (ARE, DRE core, MBS, LTR, TC-rich repeats, WRE, and WUN-motif,), transcription factor binding site (CCAAT-box, MBS, MRE, MYB recognition site, MYC, and Myb), and nitrate-responsive element (NRE) (Figure 4e,f, Table S7). These results suggest that ScNRT2.3 likely functions as a membrane nitrate transporter protein involved in LN stress responses in sugarcane roots.

### 2.5. Heterogeneous Over-Expression of ScNRT2.3 Could Enhance the Growth of Arabidopsis Under Low Nitrate Conditions

**Figure 5:**
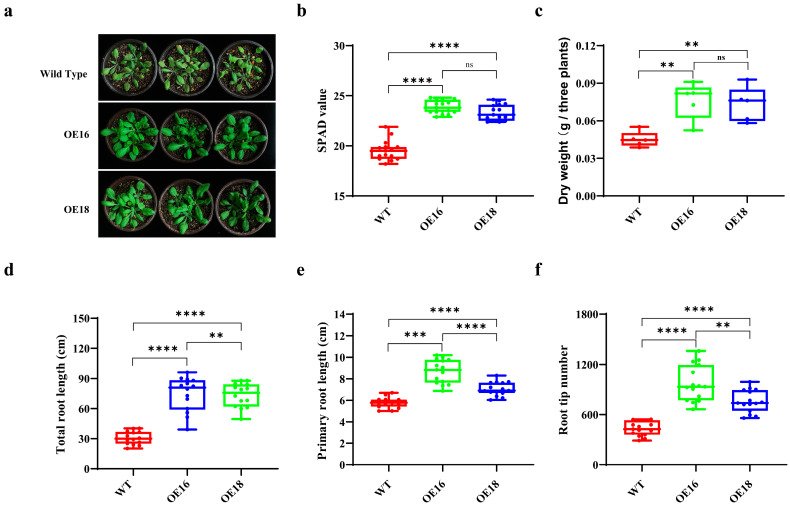
The effect of overexpressing the *ScNRT2.3* gene on *Arabidopsis* growth. (**a**) The phenotype characteristics of WT, OE16, and OE18 plants, under 0.5 mmol/L nitrate conditions. (**b**–**f**) The chlorophyll content (SPAD value), dry weight (per three plants), root tips number, total root length, and primary root length in WT, OE16, and OE18 plants under 0.5 mmol/L nitrate conditions, respectively (*n* = 5–15). The ****, ***, **, and ns on the bars represent significant differences calculated by the one-way ANOVA method with *p*-values < 0.0001, <0.001, <0.01, and >0.05, respectively.

In a bid to assess the functionality of *ScNRT2.3*, the ScNRT2.3-GFP construct was introduced into the *Arabidopsis* plants. As shown in Figure 5a,b, all two transgenic lines overexpressing *ScNRT2.3* maintained significantly higher chlorophyll relative contents (single-photo avalanche value, SPAD values) than Col-0 under low nitrate (0.5 mmol/L) conditions, which were 1.22 and 1.19 times higher than those of WT plants, respectively. In addition, the fresh weights of OE-*ScNRT2.3*-16 and OE-*ScNRT2.3*-18 were 1.68 and 1.62 times higher than those in WT plants (Figure 5c). Furthermore, both OE-*ScNRT2.3*-16 and OE-*ScNRT2.3*-18 lines presented conspicuous advantages in terms of root phenotypes, including root tip numbers, total root length, and primary root length (Figure 5d–f). The primary root lengths in both OE-*ScNRT2.3*-16 and OE-*ScNRT2.3*-18 were 1.50 and 1.22 times higher than that in WT, respectively (Figure 5d). The numbers of root tips in both OE-*ScNRT2.3*-16 and OE-*ScNRT2.3*-18 were 2.24 and 1.75 times higher than those in WT, respectively (Figure 5e). The total root lengths in both OE-*ScNRT2.3*-16 and OE-*ScNRT2.3*-18 were 2.16 and 1.68 times higher than that in WT, respectively (Figure 5f). These findings suggest that the over-expression of *ScNRT2.3* can enhance the growth of *Arabidopsis* under low nitrate conditions.

### 2.6. ScNRT2.3 Involved in the Response of Sugarcane to LN Stress Through Interaction with ScNAR2.1 Protein

**Figure 6:**
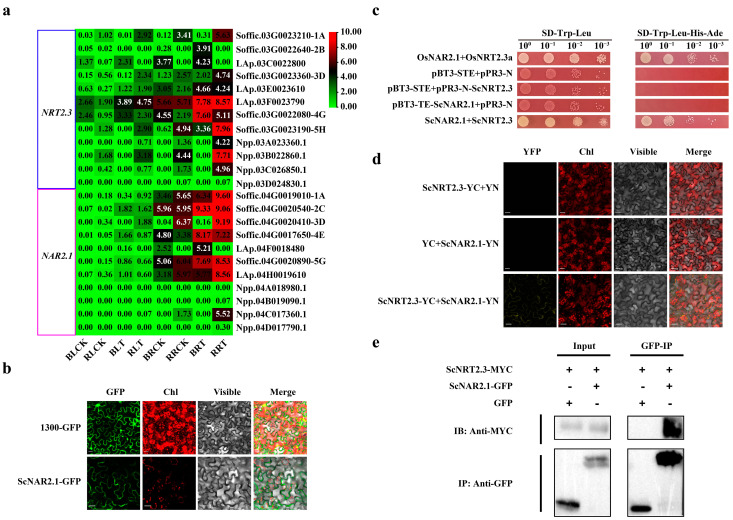
The protein–protein interaction between ScNAR2.1 and ScNRT2.3. (**a**) The expression pattern of the NRT2.3 (blue) and NAR2.1 (magenta) gene families in two NUE-contrasting sugarcane cultivars under both normal- and low-nitrogen conditions, respectively. Gene expression levels were color-coded on a change scale of 0.00 (green) to 10.00 (red). (**b**) The subcellular location of the ScNAR2.1 protein. Scale bars = 20 μm. (**c**) Y2H assays showed that ScNAR2.1 interacts with ScNRT2.3. Yeast cells co-transformed with pBT-STE-OsNAR2.1 and pPR3-N-OsNRT2.3 were used as the positive control, and pBT-STE and pPR3-N as the negative control. (**d**) BiFC assays were performed to prove the interaction of ScNRT2.3 with ScNAR2.1. The leaf epidermal cells pairwise co-transformed with ScNAR2.1-YN (ScNRT2.3-YC) plus YC (YN) were used as negative controls. Scale bars = 20 µm. (**e**) CoIP assays were conducted to confirm the interaction of ScNRT2.3 with ScNAR2.1. The immune complexes were immobilized on anti-MYC/anti-GFP magnetic beads, and the co-precipitation of ScNAR2.1 and ScNRT2.3 was examined by Western blotting using antibodies against MYC and GFP, respectively.

As shown in Figure 6a, the up-regulated expression of *NRT2.3* is accompanied by the up-regulated expression of *NAR2.1* in sugarcane, which is considered a key regulator of plant nitrogen use. Therefore, it is speculated that NRT2.3 may also interact with NAR2.1 in sugarcane. The cloning and subcellular localization analysis assays show that ScNAR2.1, a cell membrane localized protein, is also conserved, and more closely related to NAR2.1 from *S. spontaneum*, *S. officinarum*, *Sorghum*, *Z. mays*, and *O. sativa* than *Arabidopsis* and *Brassica napus* (Figure 6b and Figure S4, Table S6). The Y2H assay showed that both OsNAR2.1+OsNRT2.3 (genes from *O. sativa* were used as positive control) and ScNAR2.1+ScNRT2.3 transformants could grow on the SD-Trp-Leu-His-Ade medium, whereas the yeast co-transformants of pPR3-N and pBT-STE, pPR3-N-ScNRT2.3 and pBT-STE, and pPR3-N and pBT-STE-ScNAR2.1 could not survive on the screening medium, indicating that ScNRT2.3 is capable of interaction with ScNAR2.1 (Figure 6c). This interaction was further substantiated by a BiFC assay in *N. benthamiana*, confirming the presence of an interaction between ScNRT2.3 and ScNAR2.1 (Figure 6d). Moreover, a CoIP assay was conducted to provide in vivo validation of this interaction, with the result demonstrating the specific pull-down of ScNRT2.3-MYC by ScNAR2.1-GFP (Figure 6e). In conclusion, it can be affirmed that ScNRT2.3 interacts with ScNAR2.1.

## 3. Discussion

NUE has been a significant focus for researchers in the field of sugarcane yield production. Factors influencing NUE, including genotype, environment, and their interactions, as well as the molecular mechanisms involved in nitrogen uptake, assimilation, translocation, and remobilization, have been extensively studied over time [1,27]. In the realm of plants, comparative genomic and transcriptomic analyses have been recognized as effective methods to identify key biological processes and genes related to growth, development, yield production, and responses to various stresses [29]. Due to limitations in sugarcane reference genomes, the comparative transcriptome analysis with de novo assembly has been prevalently adopted to elucidate the divergence between different genotypes in the past decade. Through this strategy, Yang et al. [27] successfully revealed the dynamic transcriptome characteristics of two NUE contrasting genotypes, highlighting unique mechanisms and key unigenes in the high-NUE cultivar ROC22. However, unigenes derived from de novo assembly lack allelic information on the origin of DEGs from either the *S. spontaneum* or the *S. officinarum* sub-genome. Several sugarcane reference genomes have been published, including those of modern cultivars SP80-3280 [23], KK3 [24], R570 [19], and ZZ1 [25]. Studies have revealed significant variations in the chromosome structure of modern sugarcane genotypes, such as differences in the number of chromosomes derived from *S. spontaneum* and *S. officinarum*, interspecific recombination ratios from homologous or non-homologous chromosomes, and translocation ratios of *S. spontaneum* or *S. officinarum* chromosomes [19,25,26]. In the present study, 24 transcriptome sequencing libraries constructed by Yang et al. [27] were successfully re-analyzed based on the combination of the *S. spontaneum* NP-X and *S. officinarum* LA-purple genome (Figure 2 and Table S2). This analysis uniquely elucidated the contribution of the *S. spontaneum* sub-genome to the NUE of modern sugarcane cultivars (Figure 2 and Table S3). This finding aligns with the study of Zhao et al. [28], which also highlighted the role of the *S. spontaneum* sub-genome in sugar accumulation in different sugarcane cultivars with varying sugar contents. In addition, a WGCNA analysis was performed here to dissect the potential hub genes in sugarcane responses to N deficiency in ROC22 with high NUE. Although our study showed a higher BUSCOs and functional annotation rate than those reported by Yang et al. [27], the total number of genes was lower, indicating that it is necessary to improve the assembly using the genome of ROC22 in the future (Figure 1c,d).

The wild species have been considered as a valuable genetic resource, with the identification and transfer of trait-related genes providing a means to enhance crop improvement [30,31,32]. For example, in rice, the high-NUE cultivar KRIL37 carrying a small region of the *O. rufipogon* genome in the *O. sativa* L. cv Koshihikari (KH) background, was demonstrated to exhibit the more intense expression of ammonium transporter and N assimilation genes compared to KH [33]. Besides this, plant growth and development were significantly influenced by their photosynthetic capacity, and there was a close link between photorespiration and NUE. Jiang et al. [34] found a stronger photosynthetic rate in *S. spontaneum* compared to *S. officinarum*, indicating that the former may support the high NUE of ROC22 by delivering genes associated with photosynthetic pathway. The genes from *S. spontaneum*, encoding enzymes related to sucrose accumulation, also contributed to the sugar accumulation in modern sugarcane cultivars [28], although it was the main contributor to stress tolerance in modern sugarcane. Numerous studies on germplasm collection highlighted the robust adaptability and stronger tolerance to various soil types in *S. spontaneum*, such as in mountainous and hilly areas with extremely low N content, indicating that the sub-genome of *S. spontaneum* may play a vital role in the NUE of modern sugarcane cultivars [35,36,37]. Herein, genes from the sub-genome of *S. spontaneum* associated with N metabolism, carbohydrate metabolism, and photosynthetic pathways were suggested to contribute to the high NUE of ROC22 (Figure 2b,c, Table S3). These findings further support the insight that *S. spontaneum* is the main contributor to excellent stress tolerance in modern sugarcane cultivars.

WGCNA serves as a highly effective strategy for identifying the co-expression networks and exploring the associations between genes and target traits based on the expression data of RNA sequencing (RNA-Seq) [38]. It has been widely used in investigating the NUE-controlling genes in other crops. Using WGCNA analysis, several potential candidates contributing to the divergence of NUE among different tea plants were identified, including leucine-rich repeat receptor-like kinase (LRR-RLK), fasciclin-like arabinogalactan (FLA) protein, laccases (LAC), microtubule-associated protein TORTIFOLIA1 (TOR1), leucine-rich repeat receptor-like protein kinase TDR, annexin and ferritin [14]. Through WGCNA analyses, the gene *NRT2.1* and two vacuoles nitrate transporter (*CLC*) genes were identified as contributors to the high NUE of *B. napus* cv. D4-1 [29]. In *Solanum lomengena*, genes encoding the light-harvesting complex and receptor, ferredoxin-NADP reductase, catalase and WRKY33 were demonstrated to be responsible for the high NUE of AM222 using WGCNA analysis [39]. Here, genes from the *S. spontaneum* sub-genome encoding NRT2, NAR2, AMT, and GDH proteins in ROC22 played a core role in the high NUE, as revealed by WGCNA analysis (Figure 3c and Table 1). In *Arabidopsis*, *NRT2.1*, *NRT2.2*, *NRT2.4* and *NRT2.5* had been proposed to act as high-affinity nitrate transporters, playing a crucial role in nitrate uptake and accumulation in roots under low nitrate conditions [40,41,42,43]. The loss of function of *NRT2.1* and *NRT2.2* resulted in a significant growth deficiency and reduced nitrate accumulation in *Arabidopsis* roots [40]. On the other hand, NAR2.1 had been shown to function as a partner protein in the coordination of nitrate uptake with NRT2s in the roots of rice and *Arabidopsis*, respectively [42,44]. In addition, *AMT1;2* has been identified as a key high-affinity ammonium transporter, aiding rice, *Arabidopsis*, and tea plants in absorbing ammonium through their roots [14,45,46].

The high-affinity nitrate transport system (HATS) genes are crucial for plant nitrate uptake and translocation, especially in low-nitrate-availability conditions [47,48]. In *Arabidopsis*, there are eight members in this family, including seven NRT2 (*AtNRT2.1-AtNRT2.7*) and one NAR2 (*AtNAR2.1*, also named as *AtNRT3.1*) genes [42]. Among them, *AtNRT2.5* was revealed to contribute to the phloem loading of nitrate in shoots during N starvation, but was not required for growth and nitrate uptake in young plants [43]. Similar to other AtNRT2 members (except for AtNRT2.7), the high-affinity nitrate transport activity of AtNRT2.5 was also dependent on the plasma membrane protein AtNAR2.1 [42]. In rice, the HATS consisted of four OsNRT2s (*OsNRT2.1-OsNRT2.4*) and one OsNAR2 (*OsNAR2.1*) gene [44,48]. Interestingly, there are two different transcripts of *OsNRT2.3*, namely, *OsNRT2.3a* and *OsNRT2.3b*, in rice due to alternative splicing events [49,50]. The activity of HATS members in rice also relies on the presence of the OsNAR2.1 protein, with the exception of OsNRT2.3b and OsNRT2.4 [44]. Knocking down the expression of *OsNRT2.3a* resulted in a significant decrease in nitrate content in rice shoots compared to wild type plants, with accumulation in the roots, under low-nitrate-availability conditions [50]. Conversely, in *OsNRT2.3a* overexpression rice lines, there was no significant difference in nitrate accumulation and use efficiency compared to the wild type [49]. However, the overexpression of *OsNRT2.3b* significantly enhanced nitrogen accumulation and NUE in rice [49]. Furthermore, the co-overexpression of *OsNAR2.1* and *OsNRT2.3a* in rice improved grain yield and NUE compared to wild type plants [51]. In *Lotus japonicus*, knocking out the *LjNRT2.3* gene resulted in a clear-cut reduction in nitrate content in roots and a progressive slowdown in the elongation rate of lateral roots [52]. In *Triticum aestivum*, the overexpression of the *TaNRT2.5-B* gene significantly enhanced seedling growth and nitrate uptake under low nitrate conditions (0.2 mmol/L), which was also linked to the increased root elongation capacity [53]. Similarly, the overexpression of the *ScNRT2.3* gene in *Arabidopsis*, a hub gene derived from the sub-genome of *S. spontaneum* in ROC22, could also promote seedling growth and root elongation under low nitrate conditions (0.5 mmol/L), suggesting a conserved function among *NRT2.3* in response to N use. Furthermore, the assays of Y2H, BiFC, and CoIP confirmed a protein–protein interaction between ScNRT2.3 and ScNAR2.1, suggesting the presence of a conserved functional module of NAR2.1/NRT2.3 in plant nitrate uptake and utilization (Figure 6c–e). In rice, NRT2.3 was believed to play a crucial role in the cytosolic pH regulation performed by OsNRT2.3a and OsNRT2.3b, as well as in phosphorus and iron accumulation by OsNRT2.3b [49]. However, the pH-dependent histidine site in sugarcane NRT2.3 was replaced by arginine. These indications suggest a potential functional divergence between sugarcane ScNRT2.3 and rice OsNRT2.3, yet before using them for the improvement of sugarcane NUE, it is necessary to overexpress or knock out the *ScNRT2.3* in sugarcane to confirm its function.

## 4. Materials and Methods

### 4.1. Plant Materials, Culture Conditions and Nitrogen Treatment

The *Arabidopsis* plants used in this study are of the Columbia (Col) accession. The phenotype checking assays were performed as described by Alfatih et al. [54]. Firstly, seeds were placed on half-strength Murashige and Skoog (MS) medium (containing 1.0% (*w*/*v*) sucrose and 0.7% (*w*/*v*) agar) at 4 °C for 3 days. Then, the plates were placed in the incubator with an 8 h light/16 h dark photoperiod, 70% humidity, and 12,000 LX light strength. Finally, the 7-day-old plantlets were transferred to round pots containing a 3:1 vermiculite: soil mixture, under different nitrate conditions.

The 10-day-old plantlets of wild type (WT) and two overexpressing (OE16 and OE18) lines were treated with the half-strength MS nutrient solution without N source (Coolaber, Beijing, China). For nitrate treatment, 0.5 mmol/L KNO_3_ was added as the N source, and KCl was included in the solution to replace the same concentrations of potassium source. The nutrient solution was irrigated every four days.

### 4.2. Data Processing, Reads Alignment, Transcripts Assembly and Expression Level Analysis

Transcriptomic data sets were extracted from the NCBI BioProject database under accession number PRJNA533093, comprising two tissues (roots and leaves) from *Saccharum* spp. hybrids cv. ROC22 (high NUE cultivar) and *S. officinarum* cv. Badila (low NUE cultivar) under normal-nitrogen (NN) and low-nitrogen (LN) conditions [27]. To process the RNA-seq data, the clean data were obtained by removing the containing adapter and low-quality reads from the raw data via fastp (version 0.24.0) with default parameters [55], and these were aligned to a combined genome of *S. officinarum* LA-purple (2n = 8× = 80) and *S. spontaneum* NP-X (2n = 4× = 40) with orientation mode using HISAT2 (version 2.1.0) [56]. Then, the aligned reads were further assembled into transcripts using StringTie (version 1.3.3b) software in a reference-based approach [57]. All assembled transcripts were annotated against the combined genome using GFFcompare (version 0.12.6) software [58]. The gene expression levels were quantified by Transcripts Per Million reads (TPM) using the RSEM (RNA-seq by expectation maximization) module provided within the Trinity (version 2020) package [59]. Two-way BLAST (version 2.12) was used to identify the *S. spontaneum* and *S. officinarum* genes in ROC22, respectively, according to the highest value. The differential expression genes (DEGs) in roots and leaves of two sugarcane cultivars under different N treatments were characterized by DESeq2 (version 1.16.1) software with |log2 (fold change)| ≥ 1.0 and *p*-value (*p*-ajust) < 0.05, after filtering the genes with TPM less than 1 [60]. To control the false discovery rate (FDR), the obtained *p*-values were adjusted using the method described by Benjamini and Hochberg [61]. Furthermore, functional-enrichment analysis, including Gene Ontology (GO) and Kyoto Encyclopedia of Genes and Genomes (KEGG), was performed to further identify which DEGs were significantly enriched in GO terms and metabolic pathways at a Bonferroni-corrected *p*-value < 0.05 compared with the whole transcriptome background, using Goatools and Python Scipy software (version 0.6.5, Travis Oliphant, Salt Lake City, UT, USA), respectively [62].

### 4.3. Weight Gene Co-Expression Network Analysis

The weighted gene co-expression network analysis (WGCNA) was conducted using the R package on the Majorbio cloud platform (https://analysis.majorbio.com/tools/tool?cmd_id=6ibe13aupor65cgc37e96ugfgr) (accessed on 10 June 2024) in Shanghai, China [38,63]. Firstly, DEGs with a variable coefficient less than 0.2 in each of the three replicate samples were filtered out to reduce noise. Secondly, a soft-thresholding power (β power) value of 12 was chosen based on module independence and connectivity, which was selected when the fitting curve approached 0.8 for the first time. The Topological Overlap Matrix (TOM) was used for module detection using the DynamicTreecut algorithm with a minimum module size of 50, and a branch merge cut height of 0.25. Module eigengenes were then used to assess correlation with genotypes and tissues through Pearson correlation. Modules with correlation coefficients close to 0.7 (r ≈ 0.7) were selected as key modules. Finally, the coefficient network of the NAGs from *S. spontaneum* sub-genome in the key module was analyzed. The top 100 NAGs in terms of connectivity in the key module were regarded as hub genes, and the network was visualized using Cytoscape (version 3.7.2) [64].

### 4.4. Gene Cloning and Bioinformatics Analysis of the Hub Gene ScNRT2.3

According to the gene information from the reference genome of *S. spontaneum* NP-X, a hub gene (*Npp.03A023360.1*, named *NRT2.3*) with full-length CDS was identified and cloned from the high-NUE cultivar ROC22. A pair of primers was designed in the Primer premier 5.0 software for PCR cloning. To obtain the upstream promoter of the *NRT2.3*, homology-based cloning was also carried out with a specific primers pair. After being verified by sequencing, the sequence of *ScNRT2.3* and its promoter were further analyzed by various bioinformatic tools. The ExPASy (https://web.expasy.org/protparam/) (accessed on 25 July 2024) was used to predict the primary structure of ScNRT2.3. Multiple sequence alignment was performed by DNAMAN (version 6.0), and a neighbor-joining tree was constructed using MEGA 7.0 with 1000 bootstrap replicates, a passion model, and pairwise deletion [65]. The online server of PlantCARE (http://bioinformatics.psb.ugent.be/webtools/plantcare/html/) (accessed on 10 August 2024) was used to analyze the cis-acting element in the promoter of *ScNRT2.3*. The list of primers used in this assay is shown in Supplemental Table S8.

### 4.5. Subcellular Location of ScNRT2.3

A subcellular localization assay was performed according to Ling et al. [66]; the ORF of *ScNRT2.3* lacking its stop was cloned into the pCAMBIA1300-35Spro::GFP vector (primers are listed in Table S8). In the experiment on the transient expression of ScNRT2.3-GFP, the fusion construct was transferred into *Agrobacterium tumefaciens* strain GV3101 by heat shock, and the empty vector was also transferred as a positive control. Then, the positive control and fusion construct were transformed into *N. benthamiana* leaf epidermal cells and visualized using fluorescence microscope (Leica TCS SP8, Leica, Wetzlar, Germany) after 48 h infiltration, respectively. The green fluorescent protein (GFP) signals were visualized using a fluorescence microscope with GFP filter (488 nm excitation wavelength) after 36–48 h infiltration.

### 4.6. Over-Expression of the ScNRT2.3 in Arabidopsis

The *Agrobacterium tumefaciens* strain GV3101 carrying the ScNRT2.3-GFP plasmid was used to transform *Arabidopsis* Col-0 plants using the floral dip method [67]. Transgenic seedlings were confirmed by resistance screening and specific primer PCR, and self-crossed for homozygous selection up to T3 lines for analysis. The effects of the *ScNRT2.3* gene on *Arabidopsis* growth were evaluated by analyzing several parameters under low nitrate conditions (0.5 mmol/L), including leaf chlorophyll relative content (single-photon avalanche, SPAD), dry weight (DW), total root length, primary root length, and root tip number. The SPAD value of +1 leaf and root phenotype was measured with a SPAD-502 Plus (Konica Minolta Sensing, Inc., Osaka, Japan) and root analyzer (WinRHIZO system, Regent Instruments, Québec City, QC, Canada), respectively. All statistical evaluations, including analysis of variance (one way ANOVA) and significant differences, were conducted using the GraphPad Prism (version 8.0.2.263, San Diego, CA, USA) software. The statistically significant level was considered as a *p*-value  <  0.05.

### 4.7. Yeast Two-Hybrid (Y2H) Assay

To investigate the protein interaction between ScNRT2.3 and ScNAR2.1, the Y2H assay was performed as according to Yan et al. [44], and the full-length ORFs of *ScNRT2.3* and *ScNAR2.1* were inserted into the pBT3-STE and pPR3-N expression vectors, respectively. The primers are shown in Table S8. After confirmation by sequencing, the plasmids were co-transformed into the yeast strain NMY51 (Weidi, Shanghai, China) and we screened the nonselective media synthetic dropout-Trp-Leu (SD-Trp-Leu, SD-TL). Subsequently, positive colonies were cultured in SD-TL liquid medium at 30 °C overnight, followed by a 10-fold dilution with double-distilled water. The diluted cultures were then spotted onto both SD-TL and SD-T-L-His-Ade (SD-TLHA) medium. The positive controls included co-transformants of pBT3-STE-OsNAR2.1 and pPR3-N-OsNRT2.3a, while negative controls consisted of pBT3-STE/pPR3-N, pBT3-STE/pPR3-N-ScNRT2.3, pBT3-STE-ScNAR2.1/pPR3-N, and pBT3-STE-ScNAR2.1/pPR3-N-ScNRT2.3. The growth condition was observed after 48–72 h.

### 4.8. Bimolecular Fluorescence Complementation (BiFC) Assays

The BiFC assays were performed according to Ling et al. [66]; the ORF fragments of *ScNRT2.3* and *ScNAR2.1* were inserted into pCambia1300S-YN and pCambia2300S-YC to form the YN-protein and YC-protein constructs, respectively. Tobacco leaves of 4-week-old plants were co-infiltrated with the *A. tumefaciens* strain GV3101 carrying the specified construct. Following infiltration, the tobacco plants were grown in the dark for 48 h, after which the yellow fluorescent protein (YFP) signals were observed with a YFP filter (561 nm excitation wavelength) using a confocal laser scanning microscope (Leica TCS SP8, Leica, Wetzlar, Germany). The primers are shown in Table S8.

### 4.9. Co-Immunoprecipitation (CoIP) Assay

A CoIP assay was performed according to Wang et al. [68]; the ORFs of *ScNRT2.3* and *ScNAR2.1* without a stop codon were subcloned in-frame into the pBWA(V)Hs-TMVΩ-MYC and pBWA(V)Hs-TMVΩ-GFP vectors, respectively. Then, the recombinant vectors, ScNRT2.3-MYC and ScNAR2.1-GFP, carried by *A. tumefaciens* GV3101 were co-infiltrated into *N. benthamiana*. The primers used are shown in Table S8.

## 5. Conclusions

In our study, a comparative transcriptome reanalysis of two NUE-contrasting sugarcane cultivars was performed using a combination of the genomes of *S. spontaneum* and *S. officinarum*. We demonstrated that *S. spontaneum* may support the NUE of modern sugarcane by providing genes related to nitrogen and carbohydrate metabolism, photosynthesis, and amino acid metabolism, and successfully isolated *ScNRT2.3* in *S. spontaneum* from the high NUE cultivar ROC22. Interestingly, heterogeneous expression of *ScNRT2.3* in Arabidopsis plants facilitated the growth of transgenic plants under low nitrate conditions by promoting photosynthesis and root elongation. Furthermore, protein–protein interaction assays showed that a conserved protein module known as NAR2.1/NRT2.3 was also observed in sugarcane. This study has offered valuable insight into the role of the *S. spontaneum* sub-genome in establishing NUE in modern sugarcane cultivars, and has provided the candidate genes for achieving high-NUE improvements in sugarcane.

## Figures and Tables

**Table 1:** The functional annotations of candidate hub genes in the magenta module.

Module	Gene ID	Gene Name	Function Annotation
Magenta	Npp.03A023360.1	*NRT2.3*	High-affinity nitrate transporter 2.3 [*Oryza sativa*]
	Npp.03C026850.1	*NRT2.3*	High-affinity nitrate transporter 2.3 [*O. sativa*]
	Npp.04B020920.1	*AMT1;2*	Ammonium transporter 1 member 2 [*O. sativa*]
	Npp.04C017360.1	*NAR2.1*	High-affinity nitrate transporter-activating protein 2.1 [*O. sativa*]
	Npp.04C019010.1	*AMT1;1*	Ammonium transporter 1 member 2 [*O. sativa*]
	Npp.06A013790.1	*GDH2*	Glutamate dehydrogenase 2 [*O. sativa*]
	Npp.06C017840.1	*GDH2*	Glutamate dehydrogenase 2 [*O. sativa*]
	Npp.08A005010.1	*AMT1;2*	Ammonium transporter 1 member 2 [*O. sativa*]

## Data Availability

The original contributions presented in this study are included in the article/Supplementary Material. Further inquiries can be directed to the corresponding authors.

## Supplementary Materials

The following supporting information can be downloaded at: https://www.mdpi.com/article/10.3390/ijms26010226/s1.
