# Supplementary material for: Identification and Characterization of Key Genes for Nitrogen Utilization from Saccharum spontaneum Sub-Genome in Modern Sugarcane Cultivar
Source: Int J Mol Sci. 2024 Dec 30;26(1):226. doi: 10.3390/ijms26010226 (PMC11720480; doi:10.3390/ijms26010226)
Supplement: Supplementary file 1 [file ijms-26-00226-s001.zip › Supplemental Figures.pdf]

Identification and Characterization of Key Genes for Nitrogen Utilization from *Saccharum spontaneum* Sub-Genome in Modern Sugarcane Cultivar

Qianlong Hui [1], Ting Song [1], Dantong Yang [1], Qibin Wu [1,2], Jinlong Guo [1], Youxiong Que [1,2,*] and Liping Xu [1,*]

1 National Engineering Research Center for Sugarcane, College of Agriculture, Fujian Agriculture and Forestry University, Fuzhou 350002, China; huiqianlong@163.com (Q.H.); songting2024@163.com (T.S.); dantong0903@163.com (D.Y.); wqbaidqq@163.com (Q.W.); jlguo@fafu.edu.cn (J.G.)

2 National Key Laboratory for Tropical Crop Breeding, Institute of Tropical Bioscience and Biotechnology/Sanya Research Institute, Chinese Academy of Tropical Agricultural Sciences, Sanya 572024, China

* Correspondence: queyouxiong@itbb.org.cn (Y.Q.); xlpmail@126.com (L.X.); Tel.: +86-591-8385-2547 (Y.Q. & L.X.)

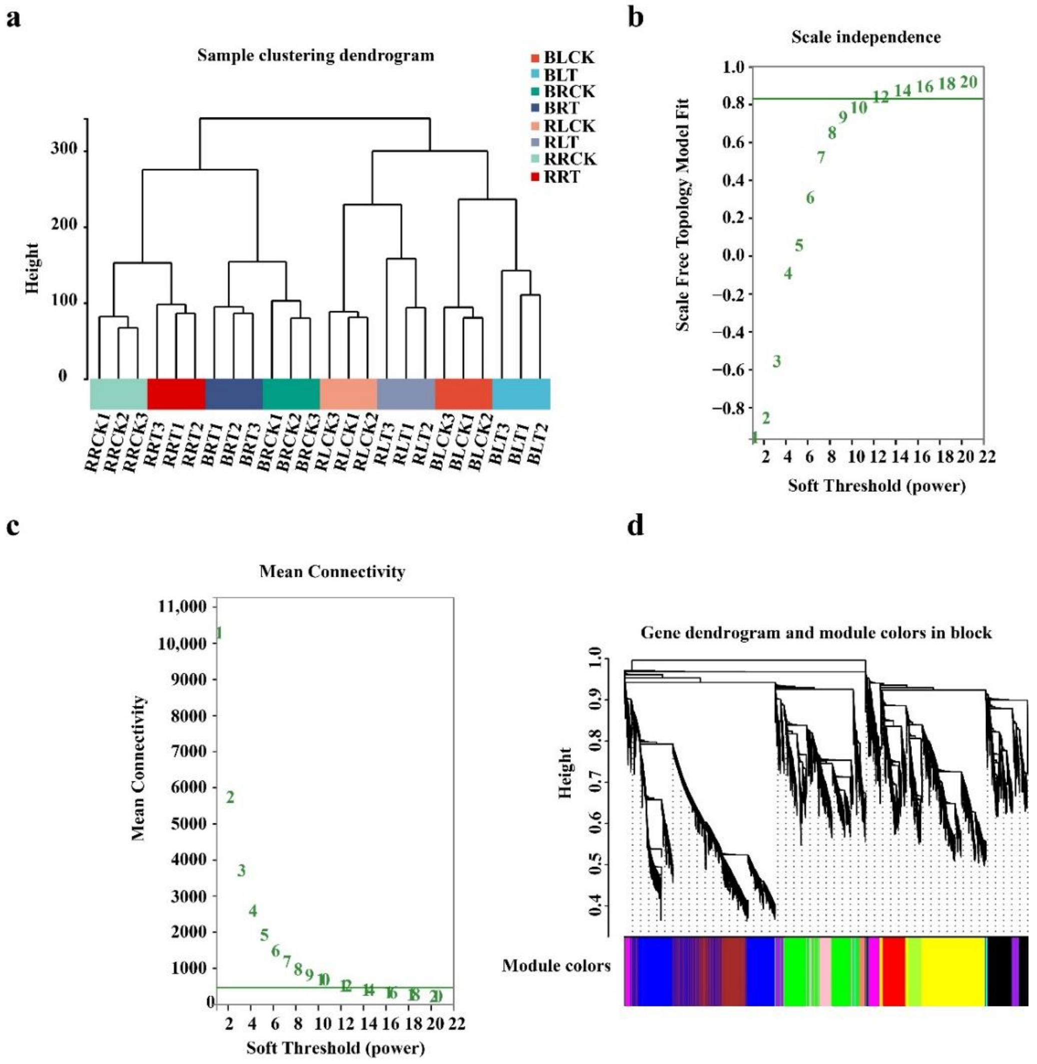

Figure S1: The weighted gene co-expression network. **(a)** The dendrogram of all 24 samples. **(b)** and **(c)** Soft threshold determination with scale independence and mean connectivity, respectively. **(d)** Module detection by gene cluster dendrogram.

1 GTGAGGGAAGAGGACACTGAGTGAGAAGCAGGCGGGCGGTGAC

44 ATGGCGGAGGTGGGGTTGAAGTCCGCGGCGATGCAGGTGGAGGCCGCCGAGGCGGCGTCC

M A E V G L K S A A M Q V E A A E A A S

104 AAGCCGCGGTTCAGGATGCCGGTGGACTCCGACAACAAGGCCACCGAGTTCTGGCTCTTC

K P R F R M P V D S D N K A T E F W L F

164 TCCTTCGCTAGGCCGCACATGAGCGCCTTCCACCTGTCGTGGTTCTCCTTCTTCTGCTGC

S F A R P H M S A F H L S W F S F F C C

224 TTCCTGTCCACCTTCGCCGCGCCGCCGCTGCTCCCCCTCATCCGGGACACGCTCGGGCTC

F L S T F A A P P L L P L I R D T L G L

284 ACGGCCACGGACATCGGCAGCGCCGGGATCGCCTCCGTGTCCGGCGCGGTCTTCGCGCGT

T A T D I G S A G I A S V S G A V F A R

344 GTCGCCATGGGCACGGCGTGCGACCTGGTGGGGCCCCGGCTGGCGTCCGCGTCCATCATA

V A M G T A C D L V G P R L A S A S I I

404 CTCCTCACCACGCCCGCCGTGTACTGCTCCGCCATCATCGACTCGCCGTCGTCCTTCCTG

L L T T P A V Y C S A I I D S P S S F L

464 CTCGTGCGCTTCTTCACGGGCTTCTCGCTGGCGTCCTTCGTGTCCACGCAGTTCTGGATG

L V R F F T G F S L A S F V S T Q F W M

524 AGCTCCATGTTCTCGCCCCCCAAGGTGGGGCTGGCCAACGGCGTCGCCGGCGGGTGGGGC

S S M F S P P K V G L A N G V A G G W G

584 AACCTCGGCGGCGGCGCCGTGCAACTAATCATGCCGCTCGTGTACGAGGCCATCCGCAAG

N L G G G A V Q L I M P L V Y E A I R K

644 ATCGGGAGCACGCCGTTCACGGCGTGGCGCGTGGCCTTCTTCATCCCGGGCCTGCTGCAG

I G S T P F T A W R V A F F I P G L L Q

704 ACGCTGTCGGCCATCGCCGTGCTGGCGTTCGGCCAGGACATGCCCGACGGCAACTACCGG

T L S A I A V L A F G Q D M P D G N Y R

764 AAGCTGCACAAGTCCGGCGACATGCACAAGGACAGCTTCGGCAACGTGCTCCGCCACGCC

K L H K S G D M H K D S F G N V L R H A

824 GTCACCAGCTACCGCGCCTGGATCCTGGCGCTCACCTACGGCTACTGCTTCGGCGTGGAG

V T S Y R A W I L A L T Y G Y C F G V E

884 CTCGCCGTGGACAACATCATCGCGCAGTACTTCTACGACCGCTTCGGCGTCAAGCTCAGC

L A V D N I I A Q Y F Y D R F G V K L S

944 ACCGCCGGCTTCATCGCCGCCAGCTTCGGGTTGGCCAACATCATCTCCCGCCCCGGCGGC

T A G F I A A S F G L A N I I S R P G G

1004 GGCCTCATGTCGGACTGGCTCTCCAGCCGGTTCGGCATGCGCGGCAGGCTGTGGGGCCTG

G L M S D W L S S R F G M R G R L W G L

1064 TGGGTGGTGCAGACCATCGGGGGCGTCCTGTGCGTTGTGCTCGGCGCCGTCGACTACTCC

W V V Q T I G G V L C V V L G A V D Y S

1124 TTCGGCGCGTCCGTGGCCGTCATGATACTCTTCTCCCTGTTCGTGCAGGCGGCCTGCGGG

F G A S V A V M I L F S L F V Q A A C G

1184 CTCACCTTTGGCATCGTGCCGTTCGTCTCCCGGAGGTCGCTGGGGCTCATCTCCGGCATG

L T F G I V P F V S R R S L G L I S G M

1244 ACCGGCGCCGGCGGCAACGTGGGCGCTGTGCTCACGCAGCTCATCTTCTTCCACGGATCC

T G A G G N V G A V L T Q L I F F H G S

1304 AAGTACAAGACGGAGACGGGGATCAAGTACATGGGGCTCATGATCATCGCCTGCACGCTG

K Y K T E T G I K Y M G L M I I A C T L

1364 CCCATCGCGCTCATCTACTTCCCGCAGTGGGGCGGCATGTTCGTGGGGCCGCGGCCCGGG

P I A L I Y F P Q W G G M F V G P R P G

1424 GCGACGGCGGAGGACTACTACAACCGGGAGTGGACAGCGCACGAGCGCGAGAAGGGGTTC

A T A E D Y Y N R E W T A H E R E K G F

1484 AACACCGCGAGCGTACGCTTTGCGGAGAACAGCGTGCGGGAGGGGGGCCGCTCAGGGAGC

N T A S V R F A E N S V R E G G R S G S

1544 CAGTCCAAGCACACTGTCCACGTCGAGTCCTCGCCGGCCGACGTGTGAAACACACACATG

Q S K H T V H V E S S P A D V *

1604 CGTACGCTAGTGCCCGTATACAATCAAAGGTCCCACGTCAGCA

Figure S2: The sequence characteristics of ScNRT2.3 in sugarcane.

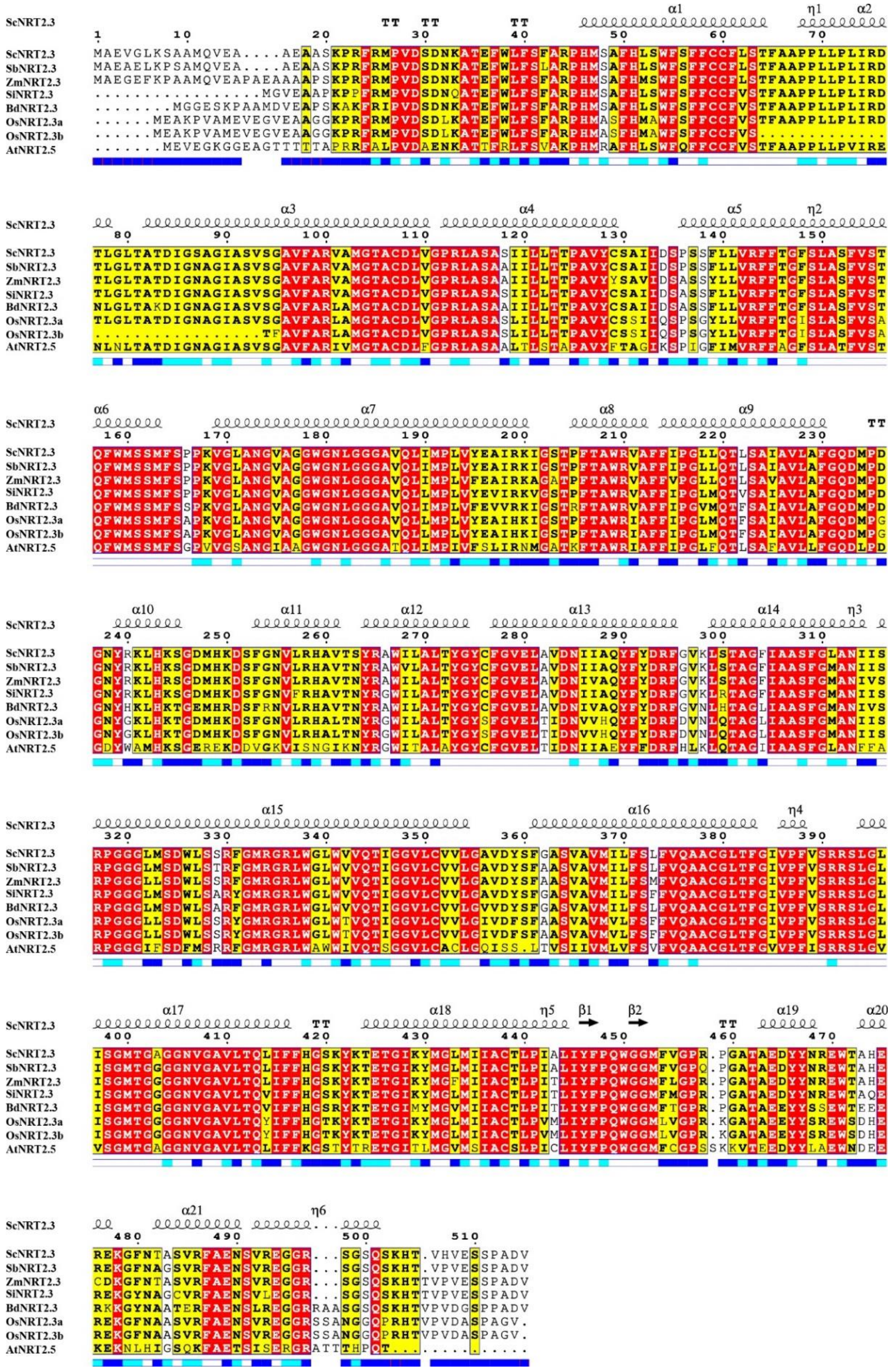

Figure S3: The multiple alignment of NRT2.3 protein sequences between sugarcane and *Sorghum*, *Zea mays*, *Oryza sativa*, *Setaria italica*, as well as *Arabidopsis*.

a

```
1   CAACCAACAGCCAGCAGAGCTGTGCCGGTCGAGCCAGGCTCCGGTAGCTAAGCTAGCGTT
61  TGGTTAAGCAAGGGATCGGTGGCCGGAGCTCGTACGTAGCGACG
105 ATGGCTCGGCACCAAAGCGTCCAGGCCTTGTCTGTGCTGGCGGTGCTTCTCTTCGCCGCC
     M  A  R  H  Q  S  V  Q  A  L  S  V  L  A  V  L  L  F  A  A
165 GCCTGCCTGCCGGCGCCGGCCGCCGCGGGGGTGCACCTCTCCACGCTGCCCAAGGCGCTC
     A  C  L  P  A  P  A  A  A  G  V  H  L  S  T  L  P  K  A  L
185 GCCGTCACCGCCTCCGCCAAATCCGGCCAAGTCCTGCACGCCGGCGTGGACTCGGTGACG
     A  V  T  A  S  A  K  S  G  Q  V  L  H  A  G  V  D  S  V  T
245 GTGACGTGGAGCCTGAACACCACCGAGCCGGCCGGCGCCGACGCCGGCTACAAGAACGTG
     V  T  W  S  L  N  T  T  E  P  A  G  A  D  A  G  Y  K  N  V
305 AAGGTGAAGCTGTGCTACGCGCCGGCGAGCCAGAAGGACCGCGGGTGGCGCAAGTCCAAC
     K  V  K  L  C  Y  A  P  A  S  Q  K  D  R  G  W  R  K  S  N
365 GACGACCTCAGCAAGGACAAGGCGTGCCAGTTCAAGGTCACCCAGCAGGCGTACGCCGCC
     D  D  L  S  K  D  K  A  C  Q  F  K  V  T  Q  Q  A  Y  A  A
425 GGCGGATCCCCCGGCAGCTTCACGTACGTCGTCGCCCGCGACGTCCCCTCGGGCTCCTAC
     G  G  S  P  G  S  F  T  Y  V  V  A  R  D  V  P  S  G  S  Y
485 TACGTGCGCGCCTACGCCACGGACGCGTCGGGCACCGAGGTGGCCTACGGCCAGACGAGC
     Y  V  R  A  Y  A  T  D  A  S  G  T  E  V  A  Y  G  Q  T  S
545 CCCGCCGCAGCCTTCGACGTCGCCGGCATCACTGGTATCCACGCGTCCCTCAAGGTCGCC
     P  A  A  A  F  D  V  A  G  I  T  G  I  H  A  S  L  K  V  A
605 GCCGGCGTCTTCTCGGCATTCTCCGTCGCCGCGCTTGCATTCTTCTTCGTCGTCGAGAAC
     A  G  V  F  S  A  F  S  V  A  A  L  A  F  F  F  V  V  E  N
665 CGCAAGAAGAACAAGTAGAGAGGCACGGCCCCGTGCGTGCGGCTGTGCCCATACATGTA
     R  K  K  N  K  *
725 ATCGTCGTGATGAGTTCTTTCCGGTCGTGACTGTCGTTGCTCCCATGGATGGATGGGTGC
785 ACGCGACTATTTTGGTGTATGTTACTTTTTCTGCAGTGTTGTGCGTGAAAATTATCAAAT
845 ACTTTGTACCGATAAGTTTCTCTGCTGCTG
```

b

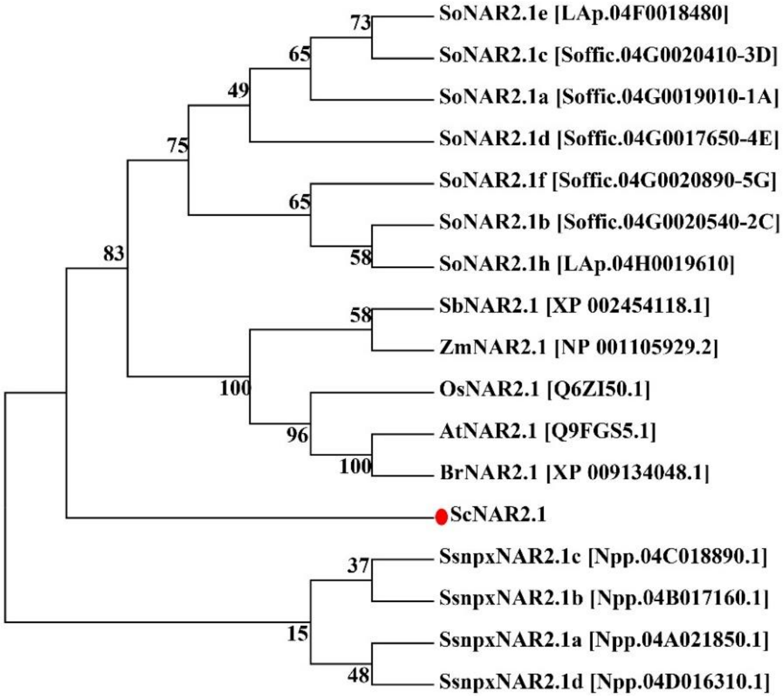

Figure S4: The sequence characteristic and phylogenetic tree of ScNAR2.1 in sugarcane. (a) The sequence characteristic of ScNAR2.1. (b) The phylogenetic tree of ScNAR2.1 protein. The Sc, So, Ss, Sb, Zm, Os, At and Br represent the *Saccharum* hybrid cultivar ROC22, *S. officinarum*, *S. spontaneum*, *Sorghum*, *Zea mays*, *Oryza sativa*, *Arabidopsis*, as well as *Brassica napus*, respectively.
